# "The Hospital" Medical Book Supplement—No. XXXVII

**Published:** 1910-12-17

**Authors:** 


### "THE HOSPITAL"
### MEDICAL BOOK SUPPLEMENT No. XXXVII

CONTENTS.

PAGE

**Medicine—**
Manual of Clinical Pathology for the General Medical Practitioner ... 351
Medical Diagnosis... ... 351

**Diseases of Children—**
The Care of Children from Babyhood to Adolescence ... 352
Medical Supervision in Schools ... 352

**Dietetics—**

Food and Hygiene ... 352

Diet and the Maximum Duration of Life ... 352

**Surgery—**
A Handbook of the Surgery of Children ... 353

PAGE

**Miscellaneous—**
A Manual of Practical Inorganic Chemistry ... 353
The Amateur Gardener's Diary and Dictionary ... 353
Laboratory Notes on Organic Chemistry for Medical Students 353
Massage Movements, including the Nauheim Exercises: An Illustrated Guide for Nurses and Masseuses ... 354
Golden Rules of Refraction ... 354
Practical Nursing for Male Nurses in the R.A.M.C. and Other Forces ... 354
The Abuse of the Singing and Speaking Voice: Causes, Effects, and Treatment ... 354

### MEDICINE.

MANUAL OF CLINICAL PATHOLOGY FOR THE GENERAL MEDICAL PRACTITIONER. By RICHARD WEISS, M.A., Ph.D., F.C.S., in collaboration with GEORGE HERSCHELL, M.D. Lond., and ANDREW CHARLES, F.R.C.S.I. London: J. and A. Churchill, 1910. Pp. 72. Price 2s. net.)

THE general practitioner, and in particular he who was educated at a time when pathological research had not attained to the importance in clinical medicine it now enjoys, will find in this short work a handy compendium of the simpler laboratory methods, such as can be carried out without complicated and expensive apparatus. Though simple the methods described are of sufficient accuracy to serve ordinary purposes of diagnosis, and what is of great importance, the directions given are easy to understand even by those who have not undergone a special training in laboratory work. The volume is confined in scope to the analyses of urine, stomach contents, fæces, and the examination of blood. Comparatively little bacteriology is introduced except in so far as recognition under the microscope of characteristic organisms is concerned, such as the Oppler-Boas or long bacillus, sarcinæ, etc. An account of a simplified Wassermann's test is given, as well as of the agglutination tests for typhoid and Malta fever. The book can be heartily recommended to those in search of a short and simple guide to the more ordinary laboratory methods.

MEDICAL DIAGNOSIS. By W. MITCHELL STEVENS, M.D., M.R.C.P., Senior Assistant Physician, Cardiff Infirmary. (London: H. K. Lewis. Pp. xi and 1571. Demy 8vo. 25s. net.)

THERE are so many books on medical diagnosis competing for popularity that it seems supererogatory, almost, to take the trouble of writing another on this, from a literary point of view, so extremely difficult subject. Dr. Stevens, we are bound to confess, brings no literary talents into requisition in discussing his subject; his style—if such a bald enumeration of points can be dignified by the name of style—is everywhere concise and terse, but lacks everything that goes to make such a book readable to those who are not forced to take it up for a special purpose. We look in vain, for instance, for any description of signs that can approach the pen pictures in the old Fagge, or, to take a more modern example, the admirable summaries which those who have had the privilege of listening to the greatest medical lecturer of the day, Krause, find indelibly fixed in their memory. But it is entirely unfair (and we readily admit our fault) to look for literary polish in a volume that deals with medical diagnosis; in a volume, moreover, that attempts to condense that enormous subject within the smallest compass. In such a book we must look to other points; serviceability, accuracy, classification, and clearness of presentation are of much more importance here than fine writing. And judging Dr. Stevens' work on these points, and at the same time comparing it with the rival manuals in the field, we must confess that we have not yet had the pleasure of reading a book which is, from a student's point of view, so ideal as this. Nor is it only the student who will appreciate the conciseness with which facts are presented here; the busy practitioner will readily give his vote in favour of a volume that combines with almost catholic fulness and a wide range of subject-matter a carefully thought-out scheme of presentation in which it is the easiest thing in the world to find any special disease or condition. As a text-book, apart from a medical classic, this work fully merits the attention of students and practitioners; the former will find it a wholly useful aid in preparing for examinations and in ward work; the latter will hold it in esteem because it is a handy and exceptionally ably devised scheme of classification which will materially help in diagnosis. There are several items which the author would do well to revise in a second edition —which we feel sure will not be long delayed. Apart from several printer's errors noted (we may instance the annoying "myostitis") and the blurring of some of the illustrations, there are some details which should be amplified. The first section, on general conditions, is wholly admirable, but the note on pigmentation is not as full as it might be; pellagra, leprosy, and after the prolonged application of Bier's treatment may be added to the list of causes. The short chapter on food poisoning is much too condensed; a few further particulars regarding the differential diagnosis of ptomaine poisoning would have been of great value to practitioners. In the note on lead poisoning no reference is made to the blood examination, which we have found of decided value in obscure cases; in that on portal pyæmia too little stress is laid on the importance of examining the prostate; in that on typhoid no mention is made of the yellow pigmentation on the palms and soles which is so distinctive a sign, according to some observers, in the early stages. We have often thought that some useful purpose would be served by a short volume on the lesser-known signs which are sometimes of use in difficult cases, and in a work of this character they ought, at least, to be mentioned. Dr. Stevens will probably agree with us that "penny-in-the-slot" diagnosis is a mistake, but every test that can be applied ought to be used where there is the slightest hesitation in pronouncing definitely with regard to a specific condition. In a future edition we therefore hope that the author will give his readers some reference to the Wassermann reaction, Quinquand's sign,

Stiller's sign, Squire's sign, Grocco's triangle, the paradoxic reflex, and some others which we do not find in the exhaustive index provided. The note on the diazo-reaction is misleading; what is described is really the modified Penzold reaction, which is almost valueless, since it is obtained in nearly every septic condition and is affected by the freshness or antiquity of the reagents employed; the real Ehrlich's reaction depends on the characteristic precipitate obtained when the red-coloured fluid is allowed to stand for a few hours.

Taken as a whole the book is a praiseworthy effort to treat a difficult subject in a manner that conduces to clearness and brevity, and as such it is eminently suitable for the practitioner's library. Considering the vast amount of useful information given, the comparatively high price of the book cannot be regarded as excessive.

### DISEASES OF CHILDREN.

The Care of Children from Babyhood to Adolescence. By Bernard Miers, M.D. With a Preface by G. F. Still, M.D. Second Edition, 1910. (London: H. Kimpton. Pp. 174. Price 1s. 6d. net; in cloth, 2s. 6d. net.)

The demand for a second edition of this book less than a year after the publication of the first one is a proof in itself that it has found favour with the public for which it is designed. In this reissue there are several additions, and the whole text has been carefully revised. The subject is treated conscientiously and thoroughly, and the most modern and up-to-date theories and methods connected with the upbringing of children are adequately described and explained. Practitioners may feel assured that this is a book which can safely and with advantage be placed in the hands of mothers and children's nurses; it is a favourable specimen of a type of book for which the demand is regular and considerable, notwithstanding a diminishing birth-rate. We have found but one point in which we are seriously displeased with the author, and that is on the subject of "croup." Otherwise the book is quite trustworthy, and, if not of any great distinction, is perhaps none the less useful as a guide to the management of babies and children.

Medical Supervision in Schools. By Edward M. Steven, M.B., Ch.M. (London: Baillière, Tindall and Cox. Covent Garden. 5s. net.)

This is the official report which Dr. Steven was commissioned to make by the Government of South Australia, on the subject of school inspection in Europe and America. It is extremely interesting for those who wish to compare the various systems now in use. For the most part the author contents himself with noting facts, leaving his readers to draw their own conclusions. We should have liked more comment on the various systems, and the opinion of so acute and so fair an observer would have been singularly useful at the present moment, when the methods adopted in London are the subject of such earnest and even acrimonious debate. It is worth while to bear in mind, however, that Dr. Steven speaks very highly of the scheme that has been elaborated by Dr. Kerr, both in Bradford and in London, and that he puts the position very fairly. On the subject of part *versus* whole time officials he is evidently reluctant to state a definite opinion; he gives the arguments for and against each side of the question and leaves it at that. There are some excellent illustrations, notably of the American methods. The author's tour of inspection embraced Germany, America, and England and Scotland, but Switzerland is only briefly discussed, while Denmark, Norway and Sweden, Belgium, and Italy are not mentioned at all. The report is therefore incomplete, but it is nevertheless very useful for purposes of comparison, and it brings out very forcibly that the best systems appear to be those in which the existing arrangements for the care and treatment of sick children are utilised as much as possible. The whole question of the establishment of independent school clinics is at present under discussion, and it is not necessary to deal with it here, even if it were possible to do so within the scope of a brief review. All that need be said is that this work should prove a valuable contribution to the literature which will be helpful in enabling us to arrive at some conclusion with reference to this important subject. Dr. Steven is to be congratulated on his report, and the publishing firm on the excellence of its presentation to the public. We only wish all reports were drawn up in this readable and attractive manner.

### DIETETICS.

Food and Hygiene. By William Tibbles, LL.D., M.D. (London: Rebman, Ltd. Second edition. Price 5s. net.)

The first edition of this little manual was published three years ago. Since then dietetics has made prodigious advances, and the author has been well advised to edit and revise a new edition of his popular work. It covers a wide range of subjects and is a popular exposition of the whole subject of dietetics. As such it is valuable and extremely useful; a book, in fact, that the practitioner may safely lend to his patient who is interested in the question of food and feeding. At the present time, when so many food faddists are about, the perusal of such a work, which gives the main facts about various matters which the laity regard as questions on which there is a wide difference of opinion among members of the faculty, is bound to prove useful. The cheapness of the new edition is a point in its favour which should not be overlooked, but we do not exaggerate when we say that its usefulness is not to be gauged by its price. It is a thoroughly sound, authoritative exposition of the subject with which it deals, and as such well suited to have a place on the shelves of the practitioner's library.

Diet and the Maximum Duration of Life. By Charles Reinhardt. (London: The Publicity Co., Ltd. 1s. net.)

This further treatise on dietetics by an exponent of the "sour milk cure," is devoted to a more general discussion of foodstuffs and diets. It contains a good deal of common-sense which might profitably appeal to the layman. Many useful points are brought out in regard to the relative values and usefulness of most of the common articles of food, but of course much stress is laid on what the author calls "lactic *bacterium* therapy," and particularly on a special brand of sour milk and cream cheese. While opinions may vary concerning this panacea, much of the advice given in the book is valuable, though there is nothing but what is well known to medical men. More attention is now being paid to the subject of diet, and it is to be hoped that a more scientific practice will prevail; certainly we may feel justified in believing that the application of the *diet absolu* or nothing but water, is more frequently called for as a preliminary treatment in many disorders.

### SURGERY.

A Handbook of the Surgery of Children. By Professor Kirmisson. Translated by J. Keogh Murphy, M.D., F.R.C.S. (London : Henry Frowde and Hodder and Stoughton. "Oxford Medical Manuals." 20s. net.)

Mr. Murphy has done good work in translating what is admittedly the best manual of the surgical diseases of children in the French language, for this volume gives a succinct and clear exposition of the theories and methods of the French school which is invaluable to those who are not in a position to read the original. The work of translation has been excellently done. Mr. Murphy's notes are everywhere elucidative, and his language is generally smooth, so that it is a pleasure to read the book. We would suggest that a companion volume giving the details of German methods and experience, on the basis of Professor Pfaundler's recent work, would be not only interesting but extremely useful for purposes of comparison. The book is especially well illustrated, the blocks being everywhere helpful and the diagrams of real utility. The work is essentially one for the practitioner, for the question of treatment receives full consideration, while the indications for operative interference are usually very clearly stated. As an instance of the excellent arrangement we may cite the chapter on appendicitis. This is one of the fullest and most exhaustive in the book, as it ought to be when one considers the importance of the subject and the difficulties of diagnosis and treatment in many cases. Special stress is rightly laid, in the paragraph on differential diagnosis, on the importance of eliminating lobar pneumonia before the case is definitely taken to be one of appendicular trouble; in children this mistake of confounding the two conditions is particularly liable to be made. Cases illustrating the difficulties of deciding between appendicitis and intestinal obstruction, intussusception, and hip disease are given, which are equally valuable to point a moral. Kirmisson does not agree that there is no medical treatment for appendicitis, and his arguments against immediate operation as a routine method of treatment are well worth careful study. With these, in the main, every experienced pediatrist will agree. He prescribes rest in bed —absolute rest, that is—the avoidance of all solids, allowing only milk or a few teaspoonfuls of iced light wine, and the application of ice to the abdomen. He prohibits the use of opium, and warns against the exhibition of purgatives. Two sentences in this admirable summary are well worth quoting : "Medical treatment of appendicitis, when prudently and methodically employed from the very beginning, nearly always gives excellent results in children. We should note that the results of such treatment are nearly always very rapid." With these dicta general practitioners who have not had the frequent opportunities of hospital surgeons for operating on cases of appendicitis in children will cordially agree. Professor Kirmisson has a deserved reputation as an orthopædist, and his remarks on deformities are therefore particularly instructive, although we confess that we do not always share his opinions as expressed in this book. The description of the treatment of clubfoot is especially good. The author differs from German orthopædists in preferring mid-tarsal arthrotomy, in long-standing cases of this condition, to tarsoclasia; the latter method, he thinks, has several dangers, among which he mentions osteomyelitis, osteitis, and fat embolism. In cases where it is necessary to operate further he chooses Nelaton's or Rydygierand's operations. Mr. Murphy's notes appended to this section on more recent methods are interesting, and it is worth while to notice that the author lays stress on the advisability of trying reduction measures in all cases before proceeding to operative interference. We lack the necessary space to deal with the other interesting points in this manual, and in conclusion we need merely add that the book is one of the finest contributions to the literature of the surgery of children's diseases which exists in the English language. It is already deservedly popular on the Continent. Mr. Murphy's translation ought to make it equally appreciated in this country.

### MISCELLANEOUS.

A Manual of Practical Inorganic Chemistry. By A. M. Kellas, B.Sc. Lond. (London : Hodder and Stoughton. 1910. "Oxford Medical Publications." Price 5s.)

This new manual should be particularly useful to medical and pharmaceutical students, as it gives much consideration to the preparation of nearly all the inorganic compounds of the British Pharmacopœia, while the scope of the work meets the requirements of the Intermediate Scientific Examination of the London University. The schemes of analysis are clear and well arranged. Laboratory manipulations are fully explained, and the teacher's work should be greatly facilitated. The several summaries will be found helpful. The sections on gravimetric and volumetric work are probably the best in the book; calculations and standard solutions are fully described, while some logarithms and other useful tables are added. In the description of Pettenkofer's method in the section on Gas Analysis, it is suggested that the breath be held during the emptying of a 5-litre bottle; surely this is something of a strain on the apnœic capacities of the student? We feel sure that this book deserves a large measure of popularity among students of chemistry, and doubtless in future editions one or two minor defects will be remedied.

The Amateur Gardener's Diary and Dictionary.

The 1911 edition of this well-known diary has been entirely re-written. A large amount of information is available in a handy form, while the schemes of work suggested month by month contain valuable hints. The little book is published at 1s. by the proprietors of "Garden Life," Hatton House, Great Queen Street, W.C.

Laboratory Notes on Organic Chemistry for Medical Students. By Paul Haas, D.Sc., Ph.D. (London : Macmillan and Co., Ltd. 1910. Pp. 128. Price 2s. 6d. net.)

The author's object in writing this short work is to provide a small book on practical chemistry adapted especially to the needs of medical students. A perusal of the volume leads to the opinion that in this he has succeeded, for, although the student must be cognisant of the main outlines of chemistry before he can hope to use the volume with understanding, the style of the author is straightforward, his explanatory matter clear and to the point, and he takes every opportunity throughout of impressing on the reader the practical bearing which the subject in hand has upon medicine. Thus the reader will realise that chemistry is not to be regarded merely as an examination subject to be got rid of as soon as possible and then forgotten, but rather as an adjunct of value to his knowledge for the fuller understanding of his clinical and other studies. The subject-matter covers and extends somewhat beyond the syllabus of the practical examination in organic and applied chemistry of London University.

Massage Movements, including the Nauheim Exercises : An Illustrated Guide for Nurses and Masseuses. New edition. (London : The Scientific Press, Ltd. Price 1s. net.)

This booklet gives the barest outlines of the art of massage; it is, in fact, little more than a list of definitions. It contains, however, a large number of diagrams and illustrations of the movements, including the Nauheim exercises. The pronunciation is given of most of the terms employed, but this is occasionally rather feeble and sometimes incorrect. A second part consists of a list of the muscles, bones, vessels, and nerves of the human body, with neat diagrams showing the position of some of them. As a first introduction to the subject this little book may be recommended to those interested in massage, but it is in no sense a text-book.

Golden Rules of Refraction. By Ernest Maddox, M.D., F.R.C.S. Third edition. (Bristol : John Wright and Sons, Ltd. Price 1s.)

It is not surprising that these neat little books of the "Gold Rules Series" should have obtained so much favour as they have done. If rightly used by those who have already mastered the groundwork of the subject they cannot fail to be most useful, containing, as they do, sound information written in each case by a recognised authority. The third edition of the volume on refraction bears this out. In a small compass, but clearly set out, is most of the knowledge necessary for the practice of refraction. As in the past, the little volume is sure to continue to be one of the most popular of the series.

Practical Nursing for Male Nurses in the R.A.M.C. and other Forces. By Major E. M. Hassard, R.A.M.C., and Mrs. A. R. Hassard. (London : Henry Frowde and Hodder and Stoughton. 1910. "Oxford Medical Publications." Pp. 339, with tables and diagrams. 3s. 6d. net.)

We must confess to having read every page of this excellent manual with the greatest interest not unmixed with profit. It is obvious that the authors have had practical experience of every condition they describe, from the many excellent hints with which their technical descriptions are interspersed. The teaching laid down is accurate, and on the whole is set forth in an attractive manner. At times there are signs of want of care in the construction of sentences, by which their meaning is rendered somewhat obscure. It must be granted, however, that in the main the authors have succeeded in producing a book which should be of the greatest utility to those for whom it was in the first event intended. While it would, of course, be impossible to mention all forms of treatment in a small volume such as the one before us, we might suggest that in a future edition some reference be made to the treatment of fractured clavicle by the padded-ring method, by which all the unpleasantness of strapping is avoided and a more satisfactory position of the fragments attained. In addition there is the advantage that the rings can be removed daily to wash the skin, and massage and move the arms. The only disadvantage to this method is that it necessitates the patient being seen daily by the medical officer, so that the rings may be kept in the right position, and a proper degree of tension maintained with them to ensure that the shoulders are held well thrown back. A little more patience in the correction of the proof-sheets of the work before us would have ensured the avoidance of a number of mis-spellings and misprints, of which the following are examples : Orygen for oxygen (p. 83), ipecachuana for ipecacuanha (pp. 130 and 192), spitoon for spittoon (p. 143), desquammation for desquamation (p. 144), sufficeint for sufficient (p. 167), amoebae for amoeba (p. 189), and medcal for medical (p. 280).

The Abuse of the Singing and Speaking Voice : Causes, Effects and Treatment. By E. J. Moure, Associate Professor to the Faculté de Médécine, Bordeaux; and A. Bouyer Fils, ex-interne des Hôpitaux, Bordeaux, Physician to the Baths, Cauterets. Translated by Macleod Yearsley, F.R.C.S., Senior Surgeon to the Royal Ear Hospital, Medical Inspector of L.C.C. Deaf Schools, etc. (London : Kegan Paul, Trench, Trübner and Co., Ltd. 1910. Pp. 130, with twelve figures in the text. Price 2s. 6d. net.)

In this little work the authors make a strong appeal for the co-operation of singing-masters and pupils with laryngologists of experience when the question arises as to which class of voice that possessed by the pupil belongs. Without the guidance of someone conversant with the anatomy of the organs that go to make up the vocal instrument, pupils are apt to find themselves studying music totally unsuited to their particular class of voice, as well as undertaking breathing exercises of a type calculated to develop the capacity of some portion of their respiratory organs which it is not desirable to increase owing to the delicate nature of their vocal cords. For example, a light operatic tenor, most of whose singing is done with the middle or head registers, has little need of diaphragmatic or abdominal respiration, and, in fact, he may do irreparable damage to his thin and fragile cords by the production of excessive blasts of air. On the other hand, a strong tenor requires a large volume of air, and it would be a mistake to impose costal respiration on such a one. By his knowledge of the anatomy of the vocal organ as a whole, including bellows, reed, and sounding-board, the laryngologist can give useful advice as to which type of breathing is best suited to the individual, and in this manner ward off many of the calamities which follow in the train of vocal abuse. The authors maintain that "no one should be admitted to study singing, and even declamation, without having passed a probationary examination in the knowledge recognised as indispensable to this class of masters," and that "conservatoires should always possess one or several laryngologists, whose care it should be to examine the pupils periodically, at the beginning, in the course of, and at the end of their studies." Used as we are to the English notation, we found it difficult to follow the foreign one. This will not, however, trouble a singer much, as he is likely to know both. The little volume contains much teaching that appears to us to be sound, and as a whole it is interesting. Our pleasure in reading this English version would have been enhanced had the style of the translator been less involved. It is irritating to come across from time to time sentences which need to be read twice or oftener before their meaning can be grasped. The following, which occurs on page 98, is an example, and, unfortunately, not the only one, of what we mean. "Thus it is that some singer, who can by watching himself, especially if he is already a little impelled to sing, conceal almost completely this or that prodromal sign of commencing fatigue, will be, by contrast, absolutely impossible to attain the same result during laryngoscopic examination." Another defect from which the volume suffers is the absence of an index.

